# Adult-Onset Neuropsychiatric Symptoms as the Presenting Feature of Xeroderma Pigmentosum Group G: A Report of a Rare Case

**DOI:** 10.7759/cureus.61645

**Published:** 2024-06-04

**Authors:** Alvee Saluja, Harsimran Kaur, Shahbaz Anees, Vibhu Mendiratta, Kiran Agarwal, Anukriti Yadav, Md Ali Osama, L. H Ghotekar

**Affiliations:** 1 Neurology, Lady Hardinge Medical College, New Delhi, IND; 2 Dermatology, Guru Harkrishan Hospital, New Delhi, IND; 3 Dermatology, Lady Hardinge Medical College, New Delhi, IND; 4 Pathology, Lady Hardinge Medical College, New Delhi, IND; 5 Internal Medicine, Lady Hardinge Medical College, New Delhi, IND

**Keywords:** neurodegeneration, neuropsychiatric manifestations, skin photosensitivity, nucleotide excision repair, ercc5 gene, xeroderma pigmentosum g

## Abstract

Xeroderma pigmentosum is a rare autosomal recessive disorder resulting in heightened cutaneous photosensitivity due to aberrant DNA repair mechanisms. Early-life developmental delay and cognitive impairment have been described in xeroderma pigmentosum cases. However, psychiatric symptoms in adulthood as the presenting feature of xeroderma pigmentosum have not been reported. We report a young adult with xeroderma pigmentosum group G presenting with prominent neuropsychiatric manifestations and evidence of neurodegeneration. The clinical, laboratory, and radiological findings, skin biopsy, and the results of the genetic testing of the patient have been described after obtaining written and informed consent. A young adult male with skin photosensitivity since infancy developed hyper-religiosity, delusions, suicidal ideations, speech hypernasality, lower limb spasticity, and cognitive impairment over the past four years. The MRI of the brain showed diffuse cerebral atrophy. The skin biopsy from bilateral cheeks showed evidence of flattening and thinning of rete ridges, pigment incontinence, and perivascular and periappendageal inflammatory infiltrate. The whole exome sequencing in ethylenediaminetetraacetic acid (EDTA) blood revealed a compound heterozygous likely pathogenic mutation in intron 13 (c.2880-2A>G (3' splice site)) and a mutation in exon 15 (c.3146del (p.Asp1049ValfsTer12)) in the ERCC5 gene suggestive of xeroderma pigmentosum group G. This case highlights that prominent neuropsychiatric features in adulthood can occur due to xeroderma pigmentosum. Thus, xeroderma pigmentosum group G should be considered as a possibility among young adults presenting with neuropsychiatric features, evidence of neurodegeneration, and early-life skin photosensitivity.

## Introduction

Xeroderma pigmentosum (XP) is a rare autosomal recessive disorder resulting from genetic defects in the nucleotide excision repair (NER) system [1]. The NER system repairs DNA lesions ranging from ultraviolet (UV) light-induced pyrimidine dimers, chemical adducts, and intra-strand cross-links [2]. Multiple proteins encoded by different genes are involved in the proper functioning of the NER system. Upon the initial recognition of DNA damage by the XPC protein complex (encoded by the XPC gene) and the XPE proteins (encoded by the DDB2 gene), the damaged double-stranded DNA helix is opened by the transcription factor TFIIH, XPB (encoded by ERCC3 gene), and the XPD proteins (encoded by ERCC2 gene). This is followed by precisely verifying and identifying the damaged DNA strand by the XPA protein (encoded by the XPA gene) followed by cleavage of the damaged DNA strand on either side by XPF (encoded by the ERCC4 gene) and XPG (encoded by ERCC5 gene) proteins followed by filling up of the defect by DNA polymerases and ligases. Mutations in eight genes that encode for the various NER proteins can result in XP, and depending on the NER protein affected, seven complementation groups (XPA to XPG) and one variant form of XP (XPV) are described [2,3]. Due to a defective DNA repair mechanism, patients with XP are at a heightened risk of developing DNA damage on exposure to UV light and other physical/biochemical agents [4]. The classical features of the disease include skin photosensitivity, which may result in severe sunburns, skin blistering, persistent erythema after minimal sun exposure, and freckle-like pigmentation over the face. Furthermore, there is an increased risk of developing cutaneous malignancies within the first decade of life [5]. Developmental delay and early-onset neurological impairment may occur in up to 20-30% of XP cases [6]. However, adult-onset neurological symptoms as a presenting feature are rare [7]. Furthermore, prominent psychiatric symptoms at the onset have not been described as a presenting feature among adult XP cases. We report the case of a young male with a history of skin photosensitivity who presented with psychiatric symptoms and progressive neurological impairment and was subsequently diagnosed with XP.

## Case presentation

**Figure 1:**
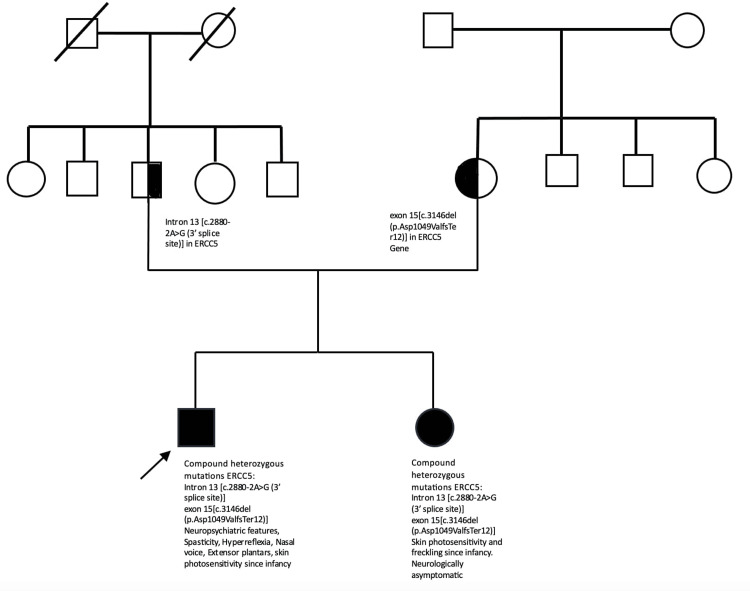
Pedigree chart depicting the inheritance pattern of XPG in the family Pedigree chart of the described case highlighting the presence of biallelic compound heterozygous likely pathogenic mutations intron 13 (c.2880-2A>G (3' splice site)) and exon 15 (c.3146del (p.Asp1049ValfsTer12)) of the ERCC5 gene in the index case and his sister. The intron 13 (c.2880-2A>G (3’ splice site)) mutation was inherited from the father, while the exon 15 (c.3146del (p.Asp1049ValfsTer12)) mutation was inherited from the mother suggesting a heterozygous carrier state in the parents.

A 25-year-old gentleman was born out of a non-consanguineous marriage via a full-term vaginal delivery and had normal milestones. At two and a half months, the mother noticed that the infant would develop skin redness and peeling on sun exposure. As a result, he avoided going out in the sun for prolonged periods. He was well till four years back when he started developing alterations in his behaviour in the form of increased religiosity, grandiose ideas, and delusions. He was taken to a psychiatrist and improved on antipsychotic medications, which he took for three months and then discontinued. At this time, he started to develop a nasal intonation while speaking. After three to four months of developing the psychiatric symptoms, he complained of lower limb stiffness and walking difficulty such that his knees would strike together while walking. Six months into the illness, he started having delusions of persecution, fearfulness, suicidal ideations, insomnia, and decreased interaction with his family members. He started forgetting which chemicals he was supposed to add while working in a rubber factory, often forgot to switch off electrical appliances, and had difficulty finding phone numbers on his phone. His symptoms gradually worsened, and antipsychotics were restarted following which his psychiatric symptoms improved. There was no history suggestive of impaired smell or vision, extraocular movement abnormality, double vision, facial numbness or deviation, hearing impairment, swallowing difficulty, decreased tongue movements, swallowing difficulty, limb weakness, or sensory loss. The patient denied abnormal limb movements, smearing of the face with food or gait imbalance, slowness in daily activities, tremors, bladder and bowel complaints, loss of consciousness, and seizures. His sister had a similar history of skin photosensitivity since infancy and freckling over the face without any neuropsychiatric manifestations (Figure 1).

**Figure 2:**
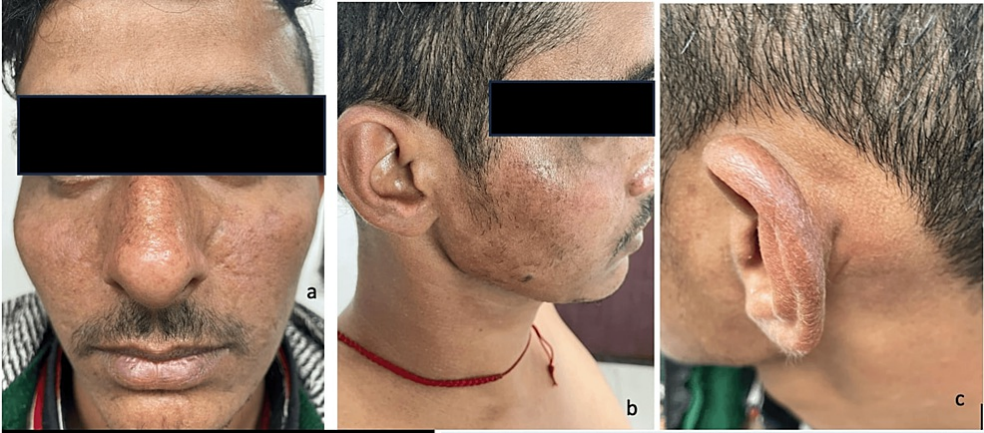
Clinical photographs of the patient demonstrating changes of chronic skin photosensitivity (2a) Clinical photograph (front view) of the patient showing patchy hyperpigmentation overlying a background of erythema, suggestive of chronic photosensitivity. (2b) Clinical photograph (side profile view) demonstrating hyperpigmented scaly plaques (sunburn) on the cheeks. Lentiginosis, poikilodermatous changes, and telangiectasias can be seen. (2c) Clinical photograph showing erythematous scaly plaque on the helix of the left ear with sparing of photo-protected retro-auricular area.

He had a normal stature with a head circumference of 52 centimetres. There were patchy brown hyperpigmented areas showing background erythema and poikilodermatous atrophy and multiple lentigines affecting the sun-exposed areas (malar area, nasal bridge, and the chin). Erythematous scaly plaques were noted over the helix with sparing of the retro-auricular area (Figure 2a-2c).

There was bilateral pes cavus. The Montreal Cognitive Assessment (MOCA) score was 15 out of 30. His voice had a nasal intonation. Bilateral lower limb clasp knife spasticity, hyperreflexia, ankle clonus, and extensor plantar responses were noted (Video 1).

**Video 1:** Clinical examination video demonstrating the neurological findings in the patient Segment 1 demonstrates bilateral lower limb spasticity. Segment 2 highlights brisk knee jerks suggestive of lower limb hyperreflexia. Segments 3 and 4 show bilateral ankle clonus and extensor plantar responses, respectively. Segment 5 demonstrates the nasal intonation in the patient's voice.

The complete hemogram, liver, renal, and thyroid function tests were within normal limits. The vitamin B12 and 25-hydroxy vitamin D3 levels were 276 picograms/millilitres and 21.05 nanograms/millilitres, respectively. The ANA, anti-dsDNA, c-ANCA, and p-ANCA antibodies were negative. The serum ferritin and ceruloplasmin levels were normal. The venous lactate, pyruvate, and ammonia levels were 18.9 mg/dl (4.5-19.8 mg/dl), 0.48 mg/dl (0.37-0.88 mg/dl), and 22 micromol/L (11-32 micromol/L), respectively. The cerebrospinal fluid (CSF) revealed four cells with a glucose of 71 mg/dl (random blood sugar: 140 mg/dl) and protein of 43.2 mg/dl. CSF Gram stain, culture and sensitivity, Ziehl-Neelsen stain, cryptococcal antigen, and India ink tests were negative. Urinary gas chromatography-mass spectroscopy and serum tandem mass spectroscopy were negative for organic acidurias (Table 1).

**Table 1:** Blood and urine investigative workup of the patient Hb: hemoglobin; TLC: total leukocyte count; DLC: differential leukocyte count; PS: peripheral smear; IU/L: international units/litre; gm/dl: gram/decilitre; mg/dl: milligrams/decilitre; mEq/L: milliequivalents/litre; pg/ml: picograms/millilitre; ng/dl: nanograms/decilitre; nmol/L: nanomole/litre; AST: aspartate transaminase; ALT: alanine transaminase; ALP: alkaline phosphatase; TC: total cholesterol; TG: triglycerides; LDL: low-density lipoprotein; HDL: high-density lipoprotein; HbA1c: glycated hemoglobin; HIV: human immunodeficiency virus; HBsAg: hepatitis B surface antigen; HCV: hepatitis C virus; ANA: anti-nuclear antigen; RA: rheumatoid antigen; c-ANCA: cytoplasmic antineutrophilic cytoplasmic antibody; p-ANCA: perinuclear antineutrophilic cytoplasmic antibody; TSH: thyroid-stimulating hormone; LDH: lactate dehydrogenase; CPK: creatinine phosphokinase; GCMS: gas chromatography-mass spectrometry; TMS: tandem mass spectrometry

S. no.	Investigation	Result	Normal reference range
1	Hb	13.8 gm/dl	13-18 gm/dl
2	TLC	4800/microlitres	4000-10,000/microlitres
3	Platelet count	196x10^3^/microlitres	150-410x10^3^/microlitres
4	PS for acanthocytes	Negative	Negative
5	AST	35 IU/L	5-35 IU/L
6	ALT	36 IU/L	5-45 IU/L
7	ALP	104 IU/L	48-128 IU/L
8	T. bilirubin	1.0 mg/dl	0.00-2.00 mg/dl
9	Urea	27 mg/dl	15-40 mg/dl
10	Creatinine	0.6 mg/dl	0.6-1.3 mg/dl
11	Sodium	144 mEq/L	136-145 mEq/L
12	Potassium	3.9 mEq/L	3.5-5.1 mEq/L
13	Calcium	10.2 mg/dl	8.6-10.2 mg/dl
14	Phosphate	3.6 mg/dl	2.5-4.5 mg/dl
15	Magnesium	2.0	1.9-2.7 mg/dl
16	TC	168 mg/dl	0-200 mg/dl
17	TG	191 mg/dl	0-150 mg/dl
18	LDL	116 mg/dl	0-130 mg/dl
19	HDL	53 mg/dl	40-60 mg/dl
20	HbA1c	5.3%	4-5.6%
21	Blood sugar-fasting	108	70-100 mg/dl
22	HIV	Non-reactive	Negative
23	HBsAg	Negative	Negative
24	IgM anti-HCV antibody	Negative	Negative
25	ANA	Negative	Negative
26	RA factor	Negative	Negative
27	c-ANCA	Negative	Negative
28	p-ANCA	Negative	Negative
29	ENA profile	Negative	Negative
30	TSH	1.54 mIU/ml	0.34-5.6 mIU/ml
31	B12	276 pg/ml	180-914 pg/ml
32	Vit. D3	21.05 ng/ml	30-70 ng/ml
33	S. ceruloplasmin	28 mg/dl	20-50 mg/dl
34	S. ferritin	21.05 ng/ml	24-336 ng/ml
35	Venous lactate	18.9 mg/dl	4.5-19.8 mg/dl
36	Pyruvate	0.48 mg/dl	0.37-0.88 mg/dl
37	Ammonia	22 micromole/L	11-32 micromole/L
38	LDH	207 IU/L	140-280 IU/L
39	CPK	236 IU/L	<171 IU/L
40	Urinary GCMS	Negative	Negative
41	Serum TMS	Negative	Negative

**Figure 3:**
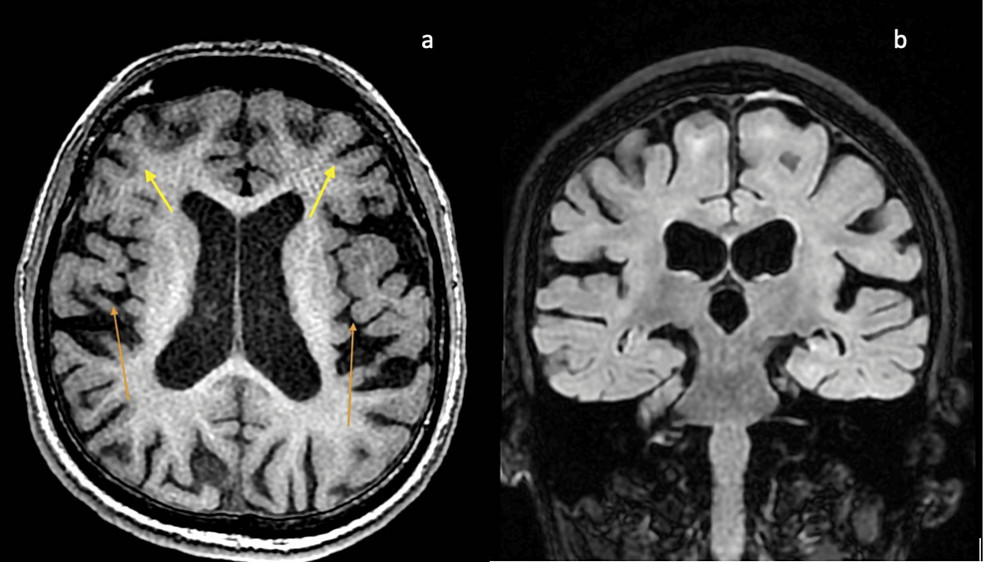
MRI of the brain images of the patient (3a) T1 axial weight image of the MRI of the brain showing diffuse cerebral atrophy disproportionate for age, predominantly in the bilateral temporal (orange arrows) and the frontal regions (yellow arrows). (3b) T1 coronal MRI of the brain demonstrating bilateral frontal and temporal atrophy disproportionate for age.

The slit-lamp examination for the Kayser-Fleischer ring was negative. Ultrasound of the abdomen was normal. The nerve conduction studies and visual evoked potential studies were normal. Pure tone audiometry suggested high-frequency sensorineural hearing loss. Diffuse cerebral atrophy, particularly in the bilateral frontotemporal regions, was noted on the MRI of the brain (Figure 3).

**Figure 4:**
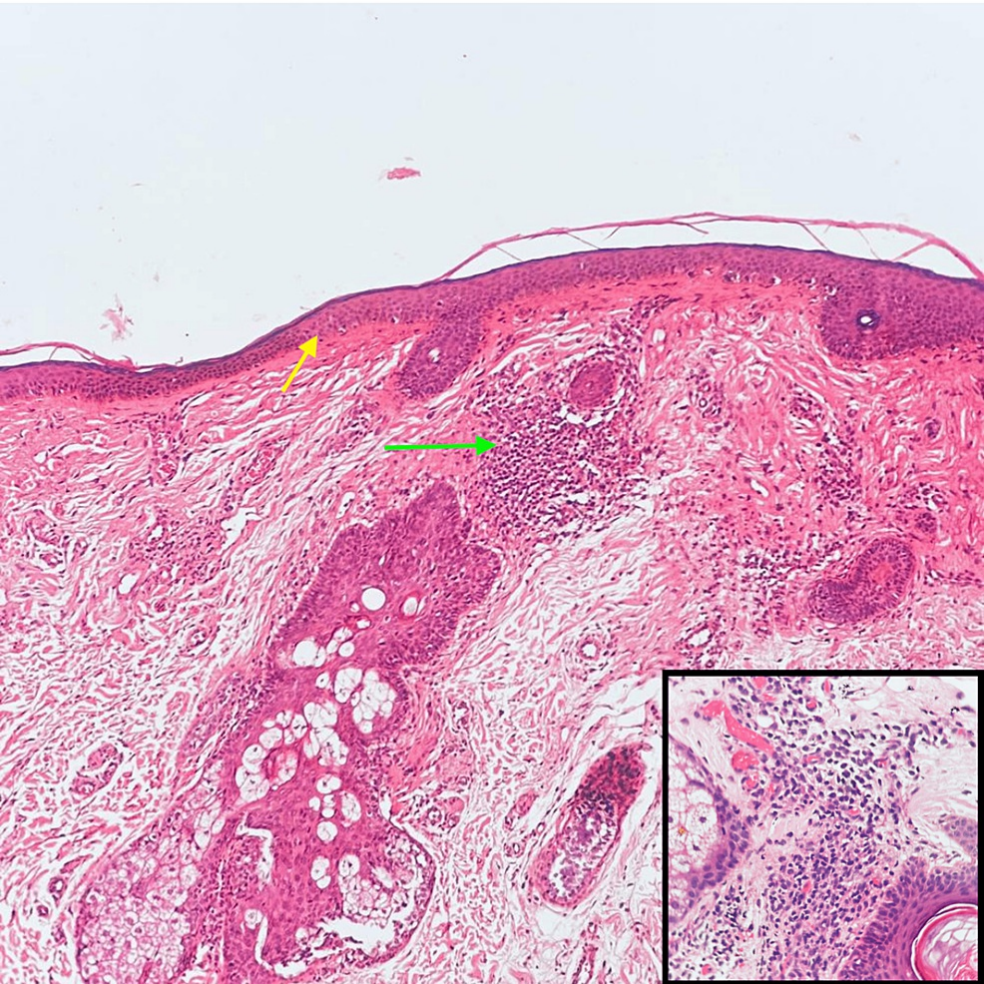
Skin biopsy from the right temporal region Skin biopsy (hematoxylin-eosin staining, 100× magnification) from the right temporal region showing flattening and thinning of the epidermis (yellow arrow). The dermis showed mild to moderate perivascular and periadnexal chronic inflammatory infiltrate (green arrow) (high-power view, hematoxylin-eosin 400×) showing the dense chronic inflammation comprising of lymphocytes and plasma cells around the appendages and blood vessels.

The skin biopsy taken from the bilateral cheeks and temporal regions showed thinning of rete ridges, focal pigment incontinence, and perivascular and periappendageal inflammatory infiltrate (Figure 4).

**Figure 5:**
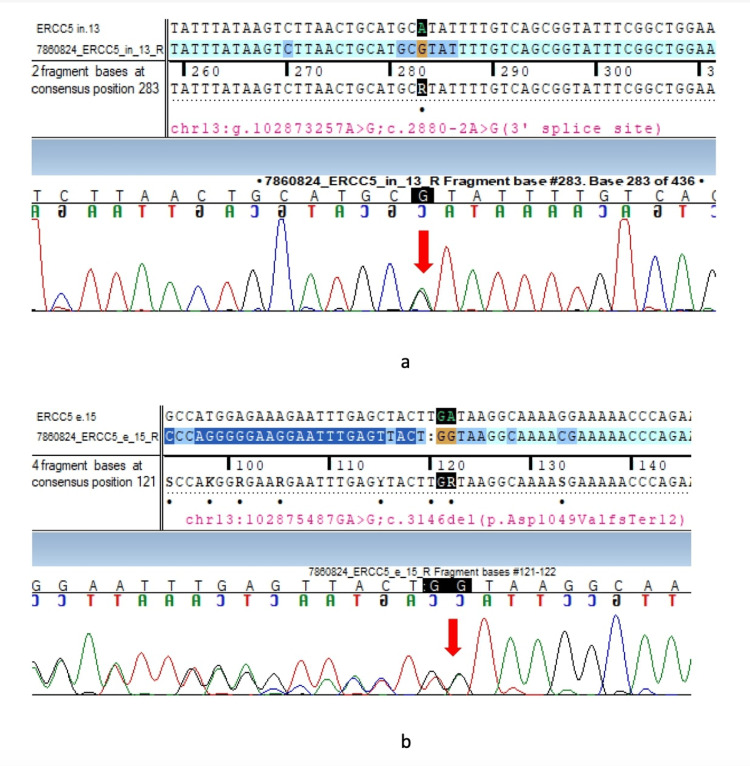
Sanger sequence chromatogram in the index case Sanger sequence chromatogram and alignment to reference sequence highlighting the likely pathogenic compound heterozygous mutation in intron 13 (c.2880-2A>G (3' splice site)) and exon 15 (c.3146del (p.Asp1049ValfsTer12)) in the ERCC5 gene (marked by the red arrow).

The whole exome and whole mitochondrial genome sequencing in EDTA blood revealed a likely pathogenic compound heterozygous mutation in intron 13 (c.2880-2A>G (3' splice site)) and exon 15 (c.3146del (p.Asp1049ValfsTer12)) of the ERCC5 gene in the index case and his sister (Figure 5).

Segregation analysis revealed the same variants in the ERCC5 gene in the heterozygous carrier state in the father (intron 13 (c.2880-2A>G (3’ splice site))) and the mother (exon 15 (c.3146del (p.Asp1049ValfsTer12))), confirming the diagnosis of XP complementation group G. The patient was started on tablets olanzapine 20 mg HS and baclofen 10 mg BD and advised physiotherapy exercises. The patient's psychiatric symptoms are controlled, and he is under follow-up.

## Discussion

Disorders of the NER system include XP, trichothiodystrophy (TTD), Cockayne syndrome (CS), De Sanctis-Cacchione syndrome, and cerebro-oculo-facio-skeletal syndrome. XP has a worldwide prevalence of one in one million individuals [8]. The disease is caused by genetic mutations in seven complementation groups (XP-A to XP-G) and one variant form (XP-V). The XP-A, XP-C, and XP-V are the most common variants comprising 30%, 27%, and 23.5% of cases, respectively, with the XP-G group comprising only 1% of all XP cases worldwide [9]. The XPG protein is a structure-specific endonuclease encoded by the ERCC5 gene and interacts with the replication protein A. During DNA repair, the XPF and XPG proteins excise the damaged DNA strand at the 5’ and 3’ ends, respectively, and are crucial proteins in the NER system [10].

Skin manifestations occur early in XPG and are usually the first to appear. Exaggerated sunburns on minimal sun exposure and mild freckling (lentigines) in sun-exposed areas are common in XPG. Due to early sunburns, photoprotective measures are instituted early, and the occurrence of skin malignancies in XPG may be lower [11]. In this case, too, skin peeling on sun exposure occurred in infancy, and there was a lack of skin malignancy (perhaps due to avoidance of skin exposure from early life). 

The neurological features that have been commonly described among XP patients are sensorineural hearing loss, microcephaly, ataxia, cognitive impairment, and peripheral neuropathy [6]. These neurological features commonly occur early, and the presence of neurological manifestations in XP portends a poorer prognosis (median age at death 29 years) [12]. In a prospective cohort of 93 patients (pediatric and adult) with XP followed up over 12 years, only eight (8.7%) patients with XP-G were identified, with seven having neurological manifestations. Similar to our case, lower limb hyperreflexia, Babinski's sign, sensorineural hearing loss, and cognitive impairment were commonly observed among the XPG group [13]. However, psychiatric manifestations were not reported. In a case report by Garcia-Moreno et al., XP-G was associated with a Huntington-like phenotype with mild apathy at the onset and overt neuropsychiatric manifestations developed later in the disease course [14]. Furthermore, prominent psychiatric manifestations at the onset have not been previously described among the 13 patients with adult-onset neurodegeneration due to mutations in the NER system [15]. 

Similar to published literature, the MRI of the brain suggested diffuse cerebral atrophy in our case [15]. Our case exhibited decreased interaction with his family members which could be attributed to apathy due to the atrophy of the bilateral frontal cortices (especially the left median frontal cortex) and the anterior cingulate gyri [16]. Hyper-religiosity has a complex neural network and can have multiple localizations such as the medial temporal lobe, hippocampus, frontal lobe, and periaqueductal grey matter [17]. The predominant atrophy of the temporal and frontal lobes might explain the hyper-religiosity in our patient. Grandiose ideation and delusions localize to the right temporal lobe (particularly the medial temporal lobe and hippocampus) and the left frontal lobe and may be due to the prominent bilateral frontotemporal neurodegeneration and atrophy in this case [18]. Articulatory disturbance with distortion of consonants and vowels with nasalization has been described in frontotemporal neurodegeneration and could be the reason for nasal speech [19]. The delayed development of neurological manifestations could be due to accruing oxidative DNA damage over time due to defective DNA repair, thus resulting in progressive neuronal loss, neurodegeneration, and brain atrophy [20]. 

## Conclusions

This case report describes psychiatric symptoms as a presenting manifestation in a patient with XPG for the first time along with highlighting the clinical manifestations of hyperreflexia, cognitive impairment, and dysarthria among adult XPG patients. Hence, neurologists should be aware of XP as a possible cause of neuropsychiatric manifestations and diffuse cerebral atrophy (due to neurodegeneration) among young adults with a history of skin photosensitivity early in life. The case attempts to highlight clinical findings that should alert the clinician to the possibility of XP so that early genetic testing and subsequent photoprotective measures can be instituted early in the disease course. 
